# Basigin can be a therapeutic target to restore the retinal vascular barrier function in the mouse model of diabetic retinopathy

**DOI:** 10.1038/srep38445

**Published:** 2016-12-05

**Authors:** Mitsuru Arima, Dan Cui, Tokuhiro Kimura, Koh-Hei Sonoda, Tatsuro Ishibashi, Satoshi Matsuda, Eiji Ikeda

**Affiliations:** 1 Department of Pathology, Yamaguchi University Graduate School of Medicine, 1-1-1 Minami-Kogushi, Ube, Yamaguchi 755-8505, Japan; 2 Department of Ophthalmology, Kyushu University Graduate School of Medical Sciences, 3-1-1 Maidashi, Higashi-ku, Fukuoka City, Fukuoka 812-8582, Japan; 3 Department of Ophthalmology, Yamaguchi University Graduate School of Medicine, 1-1-1 Minami-Kogushi, Ube, Yamaguchi 755-8505, Japan; 4 Department of Cell Signaling, Institute of Biomedical Sciences, Kansai Medical University, 2-5-1 Shinmachi, Hirakata, Osaka 573-1010, Japan

## Abstract

Despite the advance in medical technology, diabetic retinopathy (DR) is still an intractable disease which leads to the damage of retinal cells and finally the visual loss. Impairment of retinal vascular barrier triggered by an admixture of multiple inflammatory cytokines is a core of pathophysiology of DR. Therefore, the molecules involved commonly in multiple cytokines-induced impairment of vascular barrier would be the targets of curative treatment of DR. Here, we demonstrate that basigin, a transmembrane molecule expressed in neural barrier-forming endothelial cells, is the molecule essential for vascular barrier impairment which is shared by various triggers including VEGF, TNFα and IL-1β. *In vitro* data with neural microvascular endothelial cells indicated that stimulation with cytokines decreases the levels of claudin-5 in cell membranes and consequently impairs the barrier function in a manner dependent on the interaction of claudin-5 with basigin and caveolin-1. In addition, the increased vascular permeability in retinas of streptozotocin-induced diabetic mice was shown to be clearly normalized by intravitreous injection of siRNAs specific for basigin. This study has highlighted basigin as a common essential molecule for various stimuli-induced impairment of retinal vascular barrier, which can be a target for strategies to establish a curative treatment of DR.

Blood vessels in central nerve systems form the barriers, such as blood-brain barrier and inner blood-retinal barrier, to maintain the proper microenvironment for cells in brain and retina. It is generally accepted that the breakdown of this barrier function occurs and accelerates the irreversible neural damage in various neural diseases. Diabetic retinopathy (DR) is a representative disorder with breakdown of neural vascular barrier which leads to the impairment of visual acuity in a huge number of diabetic patients^1^,^2^. Dysfunction of retinal vascular barrier causes diabetic macular edema (DME), and persistent DME results in the damage of neural cells and finally the visual loss. Although a therapy targeting vascular endothelial growth factor (VEGF) has become standard for DME^3^, the specification of new therapeutic targets for DME is urgent, since the anti-VEGF therapy has several problems including the necessity of repeated intravitreous injection, possible resistance for treatment and so forth. As the triggers for impairment of vascular barrier in diabetic retinas, besides retinal hypoxia, the inflammation has been highlighted by the increase in inflammatory cytokines in retina as well as vitreous fluid of patients with DR^4^,^5^. Inflammatory process is regulated by a mixture of cytokines, and therefore the downstream molecules common for multiple cytokines, if any, would be preferable as the therapeutic targets.

Neural vascular barrier function depends on the appropriate assembly of tight junction (TJ) between endothelial cells, and pathological conditions such as inflammation and tissue hypoxia are known to impair the vascular barrier through modification of the expression of integral molecules for TJ formation. Among the TJ molecules, claudin-5 is shown to be a key molecule which confers the barrier properties on neural vascular endothelial cells^6^,^7^. As for hypoxia-triggered impairment of neural vascular barrier, we have focused our study on the changes in claudin-5 expression and demonstrated that a disintegrin and metalloproteinases (ADAMs) 12 and 17 are essential molecules for neural vascular barrier impairment under hypoxia^8^. However, no molecules responsible for inflammation-triggered disruption of neural vascular barrier have been specified.

Basigin is a transmembrane molecule which is categorized as a member of immunoglobulin superfamily. Due to a diversity of processes of its discovery, basigin has several synonyms largely depending on species in which it was specified; basigin/Gp42 in mouse, HT7/neurothelin/5A11 in chick, extracellular matrix metalloproteinase inducer (EMMPRIN)/CD147 in human and so forth^9^. In relation to neural vascular barrier, HT7 was specified and cloned by its distribution exclusively to barrier-forming neural vascular endothelial cells, suggesting its contribution to vascular barrier function^10^. However, no vascular barrier-related phenotypes could be detected in mice deficient for basigin^11^, and despite efforts of many scientists, they have not been successful in figuring out the roles of basigin in barrier properties of neural vascular endothelial cells. Here, we demonstrate that basigin works as a molecule to open the neural vascular barrier under a wide range of pathological situations including inflammation, and consequently can be an effective therapeutic target for DR.

## Results

### Basigin is required for breakdown of neural vascular barrier in various inflammatory conditions

**Figure 1:**
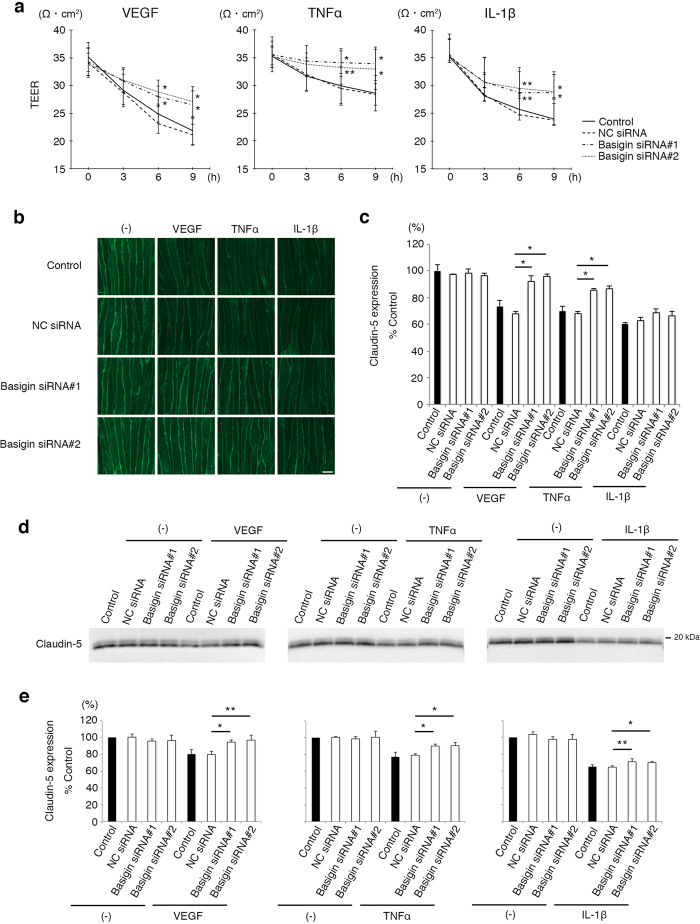
Involvement of basigin in inflammation-induced impairment of neural vascular barrier. (**a**) TEERs of bEND.3 monolayers under the stimuli of VEGF, TNFα or IL-1β. Decrease in TEERs by VEGF, TNFα or IL-1β is suppressed in cells transfected with basigin siRNAs, and the differences become significant 6 hours after stimulation. (**b**,**c**) Immunofluorescent images of claudin-5 (**b**) and their corresponding quantitative analyses for cell membrane-localized claudin-5 (**c**) in bEND.3 cells after the treatment with VEGF, TNFα, or IL-1β for 6 hours without or with the suppression of basigin expression by specific siRNAs. Differences in immunofluorescent intensities for membrane-localized claudin-5 between the cells with NC siRNA and with basigin siRNAs are statistically significant under the stimulation with either VEGF or TNFα, while not significant under IL-1β stimulation. (**d**,**e**) Western blot analyses (**d**) and their corresponding quantitative analyses (**e**) for cell membrane-localized claudin-5 isolated from bEND.3 cells through *in situ* biotinylation of cell surface molecules. Cells under the stimuli of either VEGF, TNFα or IL-1β for 6 hours are rescued from the decrease in amounts of cell membrane-localized claudin-5 by basigin siRNAs. Error bars indicate s.d. *P < 0.01; **P < 0.05; NC siRNA, non-silencing siRNA for negative control; Scale bar in (**b**), 10 μm.

In mouse brain microvascular endothelial cells, bEND.3 cells, two different sizes of basigin molecules were detected on Western blot analysis, and they were confirmed, by an analysis with tunicamycin, to be high glycosylation form of basigin (basigin-HG) and low glycosylation form of basigin (basigin-LG), respectively. (Supplementary Fig. 1a). To examine if basigin is involved in the processes of neural vascular barrier impairment by inflammation, siRNAs specific for basigin were introduced into endothelial cells. In the endothelial cells introduced with basigin-specific siRNAs, the expression levels of basigin were successfully suppressed without significant influences on claudin-5 expression, although the levels of basigin-LG were decreased predominantly and more rapidly as compared with those of basigin-HG (Supplementary Fig. 1b,c). Endothelial cells at 36 hours after the introduction of siRNAs with no significant morphological changes were processed to the experiments (Supplementary Fig. 1d). Endothelial cell monolayers were incubated with VEGF, tumor necrosis factor α (TNFα) and interleukin-1β (IL-1β) which are representative cytokines involved in the progression of various neural diseases such as DR^12^,^13^. VEGF, TNFα as well as IL-1β decreased the transendothelial electrical resistance (TEER), an index of barrier properties, of endothelial cell monolayers, while the suppression of basigin expression by specific siRNAs rescued monolayers from the fall in TEER, indicating the involvement of basigin in the inflammatory cytokines-induced impairment of vascular barrier function (Fig. 1a). Results of a confocal imaging study with quantitative analysis^8^ (Fig. 1b,c) as well as a Western blot analysis after biotinylation of cell membrane-localized molecules (Fig. 1d,e) correlated closely with those of the study on TEER, showing that the cytokines-induced disappearance of claudin-5 from endothelial cell membranes is clearly inhibited by the suppression of basigin expression. Although statistical significance was not obtained by confocal imaging study on IL-1β-stimulated endothelial cells, these data demonstrate the contribution of basigin to the inflammatory cytokines-triggered disappearance of claudin-5 from endothelial cell membranes and consequently the inflammation-induced breakdown of vascular barrier.

### Basigin combines with claudin-5 and caveolin-1, and regulates the recruitment of caveolin-1 to lipid rafts

**Figure 2:**
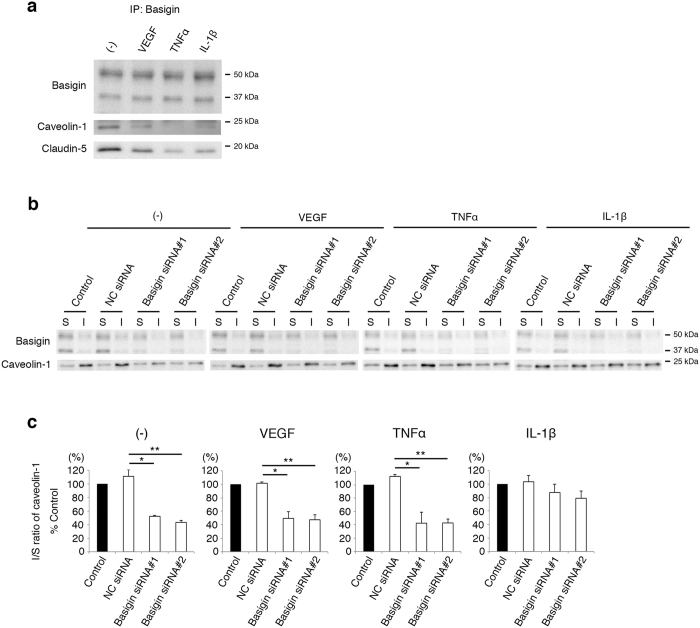
Basigin-dependent recruitment of caveolin-1 into caveolae. (**a**) Interaction of basigin with caveolin-1 and claudin-5 in bEND.3 cells. Caveolin-1 and claudin-5 are co-precipitated with basigin from bEND.3 cells under the stimuli of VEGF, TNFα or IL-1β. (**b**,**c**) Western blot analyses (**b**) and their corresponding quantitative analyses (**c**) for basigin and caveolin-1 in 1% Triton X-100-soluble and insoluble fractions. Most of basigin molecules are fractionated in the soluble fraction, while the distribution of caveolin-1 molecules alters under influence of basigin expression. Ratio of the amounts of caveolin-1 in the insoluble to soluble fractions (I/S ratio) declines by the suppression of basigin expression. Statistically, the decrease in I/S ratio of caveolin-1 by basigin siRNAs is significant under the stimulation with either VEGF or TNFα. Error bars indicate s.d. *P < 0.05; **P < 0.01; NC siRNA, non-silencing siRNA for negative control; IP, immunoprecipitation; S, soluble fraction; I, insoluble fraction.

It is also noteworthy that the suppression of basigin expression by siRNAs had no significant effects on barrier properties of endothelial cells under steady-state conditions (Fig. 1). Expression levels of basigin, both basigin-LG and basigin-HG, in endothelial cells were constitutive irrespective of cytokine treatments (Supplementary Fig. 2). In cytokine-treated endothelial cells, the suppression of basigin expression did not increase the claudin-5 mRNA levels (Supplementary Fig. 3), indicating that basigin is involved in inflammatory cytokines-triggered disappearance of claudin-5 from cell membranes by post-transcriptional mechanisms. Based on these data, we hypothesized that cytokine stimuli impair the barrier properties of endothelial cells through changes in the interaction of basigin with other molecules which may include claudin-5. It has been reported that claudin-5 localized in cell membranes of neural vascular endothelial cells is internalized by caveolae-mediated endocytosis under pathological conditions^14^,^15^,^16^. In addition, caveolin-1, an essential molecule for the assembly as well as the function of caveolae^17^,^18^, is reported to regulate the glycosylation of basigin^19^. Consistent with our expectation, immunoprecipitation study showed that basigin, caveolin-1 and claudin-5 form a comlex in endothelial cells (Fig. 2a). The amounts of caveolin-1 and claudin-5 co-precipitated with basigin were decreased by a variety of cytokine stimuli, which might reflect the disappearance of claudin-5 from cell membranes (Fig. 2a). To understand the biological significance of complex formation among basigin, caveolin-1 and claudin-5, we analyzed the subcellular distribution of basigin and caveolin-1 with focus on their recruitment to caveolae, which are assembled in the specific microdomains of cell membranes called lipid rafts. Proteins of endothelial cells were isolated into two fractions which are soluble and insoluble, respectively, in 1% Triton X-100 at 4 °C as described previously^20^,^21^. Lipid rafts are enriched in cholesterol, sphingolipid and glycolipid, and therefore the proteins within lipid rafts are concentrated in the insoluble fraction rather than the soluble fraction. Caveolin-1 was isolated predominantly in the insoluble fraction, while most of basigin was isolated in the soluble fraction (Fig. 2b). Interestingly, the ratio of caveolin-1 in the insoluble to the soluble fractions was decreased by suppression of basigin expression with siRNAs both in unstimulated endothelial cells and in VEGF or TNFα-stimulated cells (Fig. 2c). These results indicate the critical role of basigin in the recruitment of caveolin-1 to lipid rafts of endothelial cells under the physiological as well as inflammatory conditions with VEGF and TNFα, although other molecules might be also involved in the incorporation of caveolin-1 to caveolae upon IL-1β stimulation.

### Claudin-5 colocalizes with caveolin-1 under inflammation in a manner dependent on basigin

**Figure 3:**
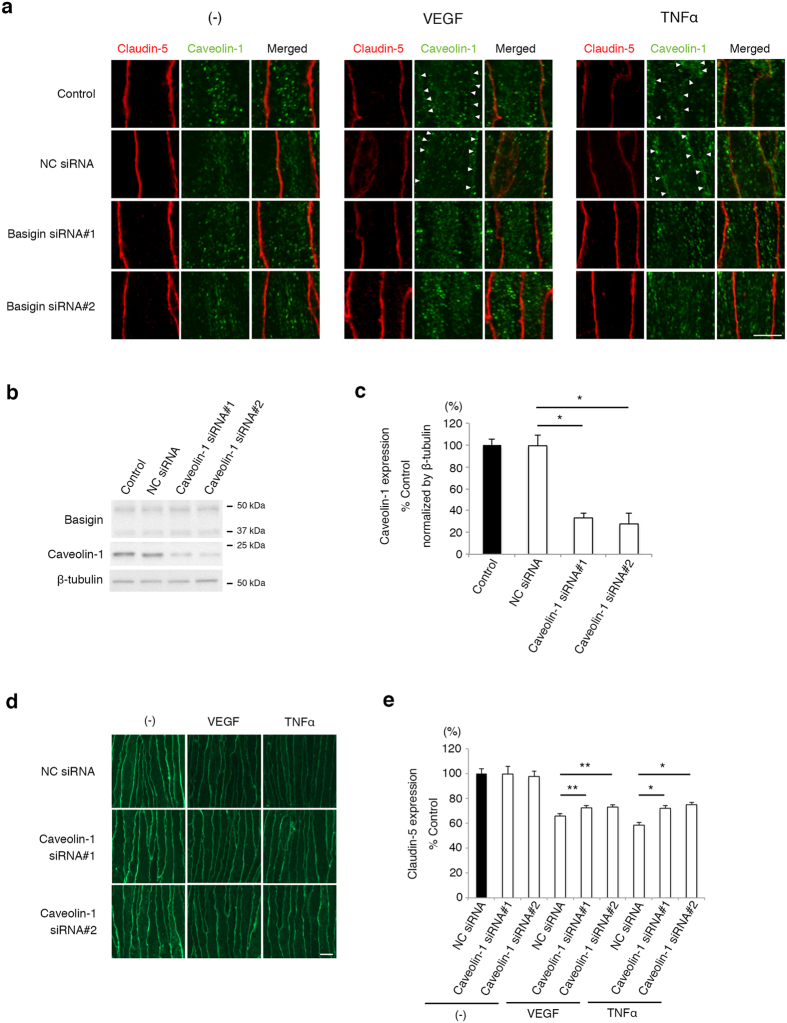
Involvement of basigin in caveolin-1-mediated internalization of claudin-5. (**a**) Immunofluorescent images for claudin-5 (red) and caveolin-1 (green). In bEND.3 cells under the stimulation of VEGF and TNFα for 6 hours, caveolin-1 molecules move to cell membranes (indicated by arrow heads). This change in the subcellular localization of caveolin-1 is blocked by basigin knockdown with siRNAs. (**b**) Western blot analyses for basigin and caveolin-1 in cells with or without the transfection of caveolin-1 siRNA. (**c**) Quantitative analyses of levels of caveolin-1 corresponding to data shown in (**b**). Introduction of caveolin-1 siRNA suppresses the expression of caveolin-1 with no influence on the basigin expression. (**d**,**e**) Immunofluorescent images (**d**) and their corresponding quantitative analyses (**e**) for cell membrane-localized claudin-5. Caveolin-1 knockdown suppresses the disappearance of claudin-5 from cell membranes in cells treated with VEGF and TNFα for 6 hours. Error bars indicate s.d. *P < 0.05; **P < 0.01; NC siRNA, non-silencing siRNA for negative control; Scale bars in (**a**) and (**d**), 10 μm.

To further reveal the biological significance of basigin-mediated incorporation of caveolin-1 into lipid rafts for the cytokines-induced disappearance of claudin-5 from cell membranes, the subcellular localization of caveolin-1 and claudin-5 in endothelial cells was analyzed by immunocytochemistry. Colocalization of caveolin-1 with claudin-5 was hardly detectable in endothelial cells under physiological condition, while their colocalization was induced by VEGF and TNFα along cell membranes (Fig. 3a). This cytokines-induced colocalization of caveolin-1 with claudin-5 was inhibited when basigin expression was suppressed with siRNAs (Fig. 3a). These results led us to hypothesize that basigin-dependent interaction between caveolin-1 and claudin-5 is a common key event underlying disappearance of claudin-5 from cell membranes upon a variety of cytokine stimulation presumably through caveolin-1-mediated endocytosis. Actually, siRNA-mediated downregulation of caveolin-1 (Fig. 3b,c) significantly attenuated disappearance of claudin-5 from cell membranes under inflammatory conditions (Fig. 3d,e), demonstrating the possible involvement of caveolin-1 in inflammation-triggered impairment of neural vascular barrier. It seems thus likely that the impairment of barrier properties of endothelial cells under inflammation is mediated by basigin-dependent interaction between caveolin-1 and claudin-5.

Our data clearly demonstrate that basigin is generally involved in breakdown of neural vascular barrier upon various cytokine stimulations. In addition, we found that basigin contributed to hypoxia-induced vascular barrier impairment as well (Supplementary Fig. 4). These findings prompted us to evaluate the availability of basigin as a therapeutic target for DR in which an admixture of inflammatory cytokines as well as, at least in part, hypoxia gives rise to pathological breakdown of retinal vascular barrier.

### Suppression of basigin expression restores the barrier function to retinal vasculature in diabetic mice

**Figure 4:**
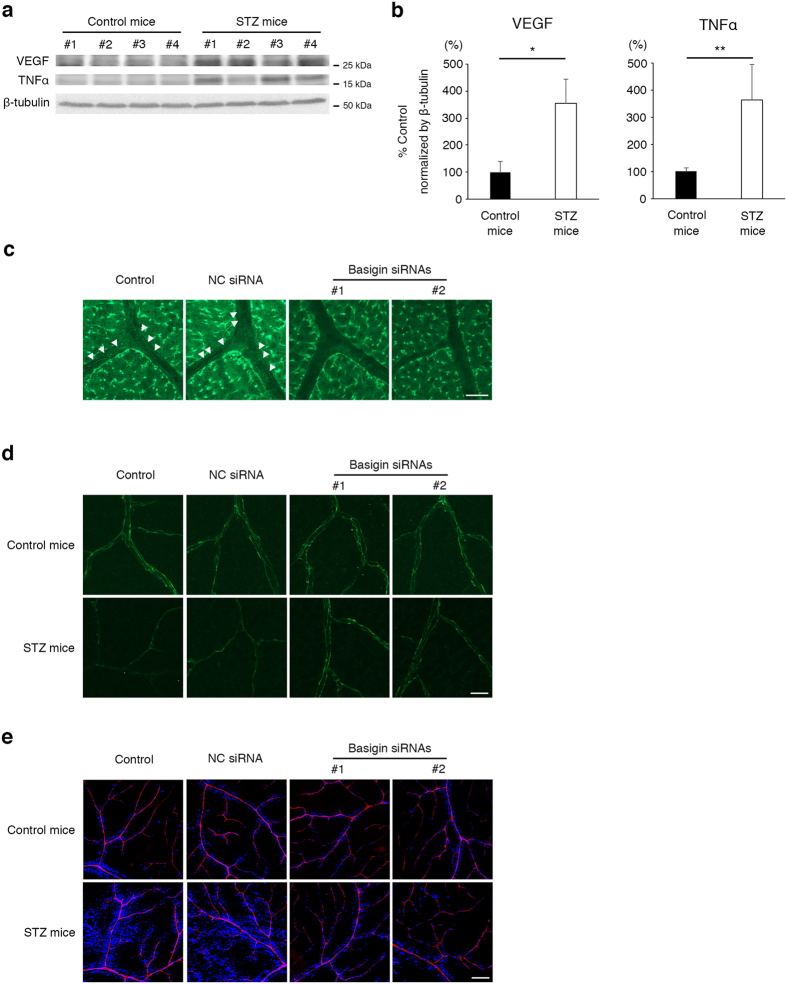
An essential role of basigin in the impairment of retinal vascular barrier function under diabetic condition. (**a**,**b**) Western blot analyses (**a**) and their corresponding quantitative analyses (**b**) for VEGF and TNFα in retinas of 4 control as well as 4 STZ-induced diabetic mice. Levels of VEGF and TNFα are significantly increased in retinas of STZ mice. (**c**) Immunofluorescent images for basigin in retinal flat mounts from mice without or with an intravitreous injection of basigin siRNA. Linear and granular signals of basigin are observed along endothelial cells in control and NC siRNA-injected mice (indicated by arrowheads in left two panels), while those signals are successfully attenuated by in basigin siRNA-injected mice (right two panels). Experiments were performed 6 times. (**d**,**e**) Immunofluorescent images for claudin-5 (**d**) and permeability (**e**) in retinal vasculature from control and STZ-induced diabetic mice without or with an intravitreous injection of basigin siRNA. Intravitreous injection of basigin siRNAs clearly rescues the retinal vasculature in STZ-induced diabetic mice from the disappearance of claudin-5 and consequently the enhanced leakage of fluorescent dye (blue). Experiments were performed 6 times. Error bars in (**b**) indicate s.d. *P < 0.01; **P < 0.05; NC siRNA, non-silencing siRNA for negative control; Scar bars in (**b**,**c**), 20 μm; Scale bar in (**d**), 100 μm.

As an *in vivo* model of DR, we used streptozotocin (STZ)-induced diabetic mice (Table 1), which showed the enhanced expression of VEGF and TNFα in retinas (Fig. 4a,b) with increased permeability of vasculature, as reported previously^22^,^23^. Basigin expression in retinal vascular endothelial cells was suppressed by intravitreous injection of siRNAs for basigin (Fig. 4c), with no detectable morphological abnormalities in retinal tissues (Supplementary Fig. 5). In retinal flat mounts of STZ-induced diabetic mice, the immunofluorescence signal of endothelial cell membrane-localized claudin-5 was diminished (Fig. 4d), and in parallel the barrier properties of retinal vasculature were impaired, which is confirmed by the increase in leakage of intracardially injected Hoechst stain H33258 (molecular mass, 534 Da) (Fig. 4e). In contrast, the intravitreous injection of basigin siRNAs clearly rescued the retinal vasculature from the decrease in immunofluorescence signals of claudin-5 on endothelial cell membranes (Fig. 4d) as well as the impairment of barrier function (Fig. 4e). These results indicate that basigin serves as a new therapeutic target to cure DME of DR.

## Discussion

Here, we demonstrate for the first time the role of basigin in neural vascular barrier which has been unknown for about 30 years since the discovery of basigin. Furthermore, our data indicate the availability of basigin as a therapeutic target for establishment new strategies to cure intractable neural diseases.

Neural homeostasis in brain and retina is maintained by vascular barrier, and it is accepted that impairment of vascular barrier function is a core of pathophysiology in various intractable neural diseases such as DR, stroke, Alzheimer’s disease and so forth^24^,^25^,^26^. In DR, which is a focus of our present study, although intravitreous injection of neutralizing antibody against VEGF is the standard therapy in clinics to control DME, the progression of DME cannot be controlled by anti-VEGF treatment in a significant number of cases. Recently, a concept of the resistance for anti VEGF therapy has been raised particularly in the field of neoplasms. Tumors are rich in various cytokines, and it has been shown that some of downstream pathways of cytokines act to attenuate the neutralization effect of anti-VEGF therapy^27^,^28^. This concept can be applied to DR, since the pathophysiology of DME is under the control of various cytokines. In addition, the resistance to anti-VEGF therapy in DR can be attributed in part to the ligand-independent activation of VEGFR-2, a receptor for VEGF^29^. Other than the anti-VEGF therapy, the intravitreous injection of TNFα inhibitor was examined in clinical trial, but it was found to be insufficient to control refractory DME^30^. Thus, the establishment of new therapeutic strategies is eagerly awaited. Previously, we specified ADAM12 and ADAM17 as the therapeutic targets to rescue the hypoxic neural tissues from breakdown of vascular barriers. Since the tissue hypoxia is known to be one of the triggers for retinal vascular barrier impairment in DR, we evaluated the availability of ADAM12 and ADAM17 as the targets to restore the vascular barrier function in retinas of STZ-induced diabetic mice. Unfortunately, we were not successful in the significant restoration of vascular barrier function in retinopathy of STZ-induced diabetic mice (data not shown), probably due to the predominant contribution of inflammatory cytokines to vascular barrier impairment in DR. These lines of evidence suggest that the targets of curative therapies for DME would be the molecules, if any, which are involved in the common downstream pathway of various pathological triggers to impair vascular barrier impairment.

In the present study, we specified basigin as a molecule which is expressed in endothelial cells and is essential for inflammation-triggered impairment of neural vascular barrier. Since basigin was shown to open the vascular barrier as a common downstream molecule of various triggers including multiple cytokines and hypoxia, basigin is expected to be a new therapeutic target for DR to restore, with high efficiency, the vascular barrier function and consequently rescue neural cells from irreversible damages. Caveolin-1 was also highlighted by our analysis as the molecule regulating neural vascular barrier through interaction with basigin and claudin-5, and therefore might be a candidate for the therapeutic target to control the function of vascular barrier. However, a past study on caveolin-1-deficient mice demonstrated that the suppression of caveolin-1 expression results in vascular hyperpermeability due to the impairments of TJ assembly^31^, while no vascular barrier-related phenotypes could be detected in mice deficient for basigin under physiological condition^11^. Therefore, basigin is considered to be preferable, rather than caveolin-1, as a target without significant side effects. Actually, in our present study, the intravitreous injection of siRNAs specific for basigin restored dramatically the vascular barrier function in retinopathy of STZ-induced diabetic mice, indicating that basigin is a promising target of curative treatment for DME. Furthermore, considering our *in vitro* data showing the general involvement of basigin in various cytokines-induced as well as hypoxia-induced breakdown of neural vascular barrier, basigin is also expected to be a therapeutic target to restore efficiently the vascular barrier with a wide range of application to various neural diseases which are still intractable in spite of the recent advance of medical technology.

## Methods

### Cell culture

A mouse brain microvascular endothelial cell line, bEND.3, was obtained from the American Type Culture Collection (Manassas, VA) and grown as a monolayer in Dulbecco’s Modified Eagle’s medium containing 4500 mg/l glucose (Sigma-Aldrich, St. Louis, MO) supplemented with 10% fetal bovine serum at 37 °C in humidified incubators with 5% CO_2_ and 95% room air. Cells were cultured in 3.5 cm plastic dishes (BD-Falcon, Franklin Lakes, NJ) coated with 5 μg/cm^2^ rat collagen (Roche, Mannheim, Germany) or in 3.5 cm glass bottom dishes (MatTek Corp, Ashland, MA) coated with 5 μg/cm^2^ human fibronectin (Wako, Osaka, Japan). Experiments using plastic dishes and glass bottom dishes were performed in 7 days and 5 days after confluency, respectively. For stimulation with cytokines, mouse recombinant protein of vascular endothelial growth factor (VEGF) (Wako), tumor necrosis factor α (TNFα) (R&D Systems, Minneapolis, MN), or interleukin-1β (IL-1β) (R&D systems) was added into cultured medium to the final concentration of 50 ng/ml, 50 ng/ml and 10 ng/ml respectively. To suppress the glycosylation, 2.5 μg/ml tunicamycin (Sigma-Aldrich) was added to the medium 24 hours prior to the start of experiments.

### Animals

Male C57BL/6 N mice (7 week old) were obtained from Chiyoda Technol Co. (Tokyo, Japan). This study was approved by the Institutional Animal Care and Use Committee (IACUC) in Yamaguchi University, and all experimental procedures were performed according to the Rule for the Care and Use of Laboratory Animals in Yamaguchi University and The Law (No. 105), Notification (No. 88) and Guideline (No. 71) of the Government.

### Immunocytochemistry of cultured cells

Cultured cells were fixed with 100% methanol for 5 minutes at room temperature. After washing with phosphate-buffered saline (PBS), they were incubated with 10% non-immune goat serum (Thermo Fisher Scientific, Waltham, MA) for 30 minutes at room temperature to block nonspecific binding of antibodies. Then, they were reacted with rabbit polyclonal antibody against claudin-5 (1/25 dilution; Thermo Fisher Scientific) or mouse monoclonal antibody against caveolin-1 (1/200 dilution; Novus Biologicals, Littleton, CO) at 4 °C overnight. After washing with PBS, they were incubated with Alexa Fluor 488 goat anti-rabbit immunoglobulin G (IgG) (1/200 dilution; Thermo Fisher Scientific) for simple staining of claudin-5, or with Alexa Fluor 488 goat anti-mouse IgG and Alexa Fluor 568 goat anti-rabbit (1/200 dilution each; Thermo Fisher Scientific) for double stainings of caveolin-1 and claudin-5 at room temperature under light protection for 1 hour. After washing with PBS, they were mounted in fluorescence mounting medium (Dako, Glostrup, Denmark), and observed under a Zeiss LSM5 Pascal laser confocal microscope (Carl Zeiss, Jena, Germany). A quantitative analysis of the fluorescent intensity of claudin-5 on cell membranes was performed according to the methods described in our previous report^8^. In brief, 3 fields were randomly photographed and 3 straight lines were drawn on each photograph. Then, fluorescence intensities at the points of cell membranes intersected with drawn straight lines were quantified using the software pre-installed in LSM Pascal. The average of fluorescent intensities was calculated as the level of claudin-5 on cell membrane for each monolayer. All experiments were performed independently in triplicate.

### Transendothelial electrical resistance

Electrical resistance of a bEND.3 monolayer at confluent state on collagen-coated 0.9 cm^2^ inserts of 0.4 μm pore size (BD-Falcon) was measured using Millicell ERS Voltohmmeter (Millipore, Billerica, MA) as described previously^8^,^32^. We calculated the values of transendothelial electrical resistance (TEER) were calculated by subtracting the resistance of blank inserts without cells and multiplying the subtracted values by the surface areas of inserts. Each experiment was performed individually 9 times.

### Western blot analysis

Cells were lysed in 100 μl of PBS containing 0.5% Triton X-100, 1% sodium dodecyl sulfate (SDS) and Halt™ Protease and Phosphatase Inhibitor Cocktail (Thermo Fisher Scientific). Mouse retinas were homogenized with 50 μl of RIPA buffer (Wako) supplemented the same cocktail inhibitors using BioMasher II (nippi, Tokyo, Japan). After incubation on ice for 15 minutes, the lysates were centrifuged at 15000 rpm for 15 minutes at 4 °C. Protein concentrations of the supernatant were determined with Protein Assay Kit II (BioRad, Hercule, CA) and FlexStation3 plate reader (Molecular Devices, Sunnyvale, CA). After addition of Laemmli sample buffer (BioRad) supplemented 5% 2-mercaptoethanol (Sigma-Aldrich), the samples were boiled. Then, aliquits of samples containing 10 μg and 100 μg of protein from bEND.3 cells and retinas, respectively, were loaded on 12.5% polyacrylamide gels respectively. Samples were separated by SDS-polyacrylamide gel electrophoresis (SDS-PAGE) and transferred to polyvinylidene difluoride membranes, Immobilon P membranes (Millipore). Membranes were incubated at room temperature for 1 hour in Tris-buffered saline with 0.1% Tween 20 (TBS-T) containing 5% skim milk for blocking. Then, they were reacted with rabbit polyclonal antibody against basigin (1 μg/ml; generated by Scrum, Tokyo, Japan), rabbit polyclonal antibody against claudin-5 (1/300 dilution; Thermo Fisher Scientific), mouse monoclonal antibody against caveolin-1 (1/1000 dilution; Novus Biologicals), goat polyclonal antibody against VEGF (1/200 dilution; R&D Systems), mouse monoclonal antibody against TNFα (1/500 dilution; Abcam, Cambridge, MA) or rabbit monoclonal antibody against β-tubulin (1/1000 dilution; Cell Signaling Technology, Beverly, MA) at 4 °C overnight. After the wash with TBS-T, they were incubated with horseradish peroxidase (HRP) conjugated goat anti-rabbit IgG and goat anti-mouse IgG (1/1000 dilution; Dako) at room temperature for 1 hour. For the detection of VEGF and TNFα, membranes were incubated in streptavidin-HRP solution (1/1000 dilution; Dako) at room temperature for 1 hour after the reaction with biotinylated rabbit anti-goat IgG or rabbit anti-mouse IgG (1/1000 dilution; Dako) at room temperature for 1 hour. They were reacted with Amersham ECL start or ECL prime (GE Healthcare, Uppsala, Sweden) according to the manufacturers’ instructions. Then chemiluminescence was detected using LAS-1000 (Fujifilm, Tokyo, Japan). Quantitative analysis of the intensities of chemiluminescence was carried out using Image J software (National Institutes of Health, Bethesda, MD). All values were normalized to β-tubulin. Experiments were performed independently, at least, 3 times.

### Biotinylation of cell surface molecules

Cultured cells were biotinylated by incubating them at 4 °C for 1 hour with 500 μl of PBS containing 0.5 mg/ml EZ-Link^TM^ Sulfo-NHS-SS-Biotin (Thermo Fisher Scientific). Then they were scraped in lysis buffer containing cocktail inhibitors as described above. After incubation at 4 °C for 15 minutes, lysates were centrifuged at 15000 rpm for 15 minutes at 4 °C, and the supernatants were incubated in 40 μl of MagnaBind Streptavidin beads (Thermo Fisher Scientific) at 4 °C for 1 hour. Beads were boiled with 40 μl of Laemmli sample buffer, and then 10 μl of the supernatant was loaded on polyacrylamide gels to be processed for Western blot analysis. Experiments were performed independently in triplicate.

### Immunoprecipitaion

To 40 μl of each cell extract, 460 μl of PBS containing cocktail inhibitors was added to dilute the surfactant. The amount of protein in each sample was within a range from 100 to 150 μg. A sample was reacted at 4 °C overnight with 5 μg of goat polyclonal antibody against basigin (Santa Cruz Biotechnology, Paso Robles, CA) or 2.5 μg of mouse monoclonal antibody against caveolin-1, and immune complexes were collected by incubating at 4 °C for 3 hours with 30 μl of Protein G Sepharose 4 Fast Flow (GE Healthcare). Beads were boiled with 30 μl of Laemmli sample buffer, and 20 μl of the supernatant was applied on polyacrylamide gels to be processed to Western blot analysis. HRP-conjugated rat anti- mouse IgG Mouse Trueblot® and HRP-conjugated mouse anti-rabbit IgG Rabbit Trueblot® (1/1000 dilution; Rockland, Gilbertsville, PA) were used as secondary antibodies. Each experiment was performed independently in triplicate.

### Transfection of small interfering RNA (siRNA)

Silencer® Select Negative Control #1 siRNA as well as siRNAs specific for basigin (ID: s63099 and s63100, defined as basigin siRNAs #1 and #2) and caveolin-1 (ID: s63423 and s63424, defined as caveolin-1 siRNAs #1 and #2) were purchased from Thermo Fisher Scientific. Transfection of siRNAs was performed using Lipofectamine RNAiMAX (Thermo Fisher Scientific) and Opti-MEM I (Thermo Fisher Scientific) according to the manufacturers’ instructions. Final concentration of siRNAs was 10 nM. Transfection of siRNAs was performed 36, 48 and 60 hours for basigin siRNAs as well as 48 hours for caveolin-1 siRNAs before the start of experiments.

### Isolation of soluble and insoluble fractions

Cultured medium of cell monolayers was changed to ice-cold PBS and kept at 4 °C for 5 minutes to block endocytosis. Then the solution was replaced with 300 μl of ice-cold PBS containing 1% Triton X-100 supplemented protease inhibitor cocktails. After the the incubation at 4 °C for 30 minutes under rotation at 70 rpm, the solution was collected as the soluble fraction. As the insoluble fraction, the remaining cellular components were dissolved in 100 μl of the above-mentioned lysis buffer containing cocktail inhibitors. After being boiled in Laemmli sample buffer, equal amount of the soluble as well as the insoluble fractions were separated by SDS-PAGE and subjected to be processed for Western bloting as described above. Each experiment was carried out independently in triplicate.

### Diabetic animals

Body weights (BW) and blood sugar levels (BS) of mice were measured using ACCU-CHEK Aviva Nano (Roche) after a fasting for 4 hours. To induce the diabetic state, streptozotocin (STZ; Sigma-Aldrich) dissolved in 0.05 M citrate buffer (pH 4.5) was injected intraperitoneally once into mice (150 mg/kg). Control mice received an injection of equal amount of citrate buffer. At day 4 after the injection of STZ, BW and BS were measured again after a fasting for 4 hours. Mice were considered to be the state of diabetes when BS exceeded 300 mg/dl. All the experiments were performed with mice at day 7 after injection of STZ when angiogenic hyperpermeable vessels had not yet appeared^33^.

### Intravitreal injections of siRNAs

Intravitreal injections were carried out according to the procedure described in our past report^13^. Mice, which were confirmed to be in the diabetic state at day 4 after STZ injection, were deeply anesthetized by an intraperitoneal injection of sodium pentobarbital. Then 1 μl of a mixture containing 5 μM siRNA was injected into the vitreous cavity using a 32-gauge needle on a Hamilton syringe. The needle was inserted just posterior to the limbus. Mice with complications caused by intravitreal injection such as vitreous hemorrhage, obvious endophthalmitis and ophthalmatrophia were excluded from analyses.

### Permeability assay of retinal vasculature

To evaluate the retinal vascular permeability, 500 μl of PBS containing 100 μg/ml Hoechst stain H33258 (molecular mass, 534 Da; Sigma-Aldrich) and 1 mg/ml tetramethylrhodamine-conjugated lysine-fixable dextran (molecular mass, 10,000 Da; Thermo Fisher Scientific) were injected into the left ventricle according to the procedures described previously^7^,^8^,^32^. After the injection of fluorescent dyes, eyes were enucleated and immediately fixed in 4% paraformaldehyde (PFA) for 15 minutes at room temperature under light protection. Then, retinal flat mounts were prepared as described previously^8^. They were mounted in fluorescent mounting medium and observed under a Zeiss LSM510 META laser confocal microscope (Carl Zeiss). Experiments were performed independently, at least, 3 times.

### Immunohistochemistry of retina

After fixation of enucleated eyes in 4% PFA at 4 °C for 2 hours under light protection, retinas were isolated and placed in PBS containing 10% goat serum supplemented 1% Triton X-100 at room temperature for 1 hour to block nonspecific binding of antibodies. Then the retinas were reacted with rabbit polyclonal antibody against claudin-5 (1/50 dilution; Thermo Fisher Scientific) or basigin (5 μg/ml; Scrum) at 4 °C overnight. After washing with PBS containing 0.1% Tween 20, they were incubated with Alexa Fluor 488 goat anti rabbit IgG (1/200 dilution; Thermo Fisher Scientific) at room temperature for 3 hours under light protection. Finally, retinal flat mounts were made by radial incision, and mounted in fluorescent mounting medium. The flat mounts were observed using a confocal microscopy LSM Pascal. Each experiment was performed independently, at least, 3 times.

### Statistical analyses

Variance of the groups to be compared with each other was analyzed using *F*-test. Then, Student’s *t*-test was applied for analyses between the groups with equal variance, while Welch’s *t*-test for analyses between the groups with unequal variance. Differences were considered statistically significant at *P* < 0.05. All data were presented as mean ± standard deviation (s.d.).

## Additional Information

**How to cite this article**: Arima, M. *et al*. Basigin can be a therapeutic target to restore the retinal vascular barrier function in the mouse model of diabetic retinopathy. *Sci. Rep.* **6**, 38445; doi: 10.1038/srep38445 (2016).

**Publisher's note:** Springer Nature remains neutral with regard to jurisdictional claims in published maps and institutional affiliations.

## Supplementary Material

Supplementary Information

## Figures and Tables

**Table 1:** Body weights and blood-sugar levels of mice before and at day 4 after the injection of STZ.

		Non-diabetic mice	STZ-induced diabetic mice
Before	BW (g)	21.09 ± 1.27	21.58 ± 1.22
	BS (mg/dl)	143.88 ± 21.70	142.51 ± 14.69
Day 4	BW (g)	22.26 ± 1.26	19.26 ± 1.11*
	BS (mg/dl)	154.88 ± 18.00	432.00 ± 57.40*

Data are presented as mean ± s.d. (*n* = 43). *P < 0.01; STZ, streptozotocin; BW, body weights; BS, blood-sugar level.
