# Supplementary material for: Basigin can be a therapeutic target to restore the retinal vascular barrier function in the mouse model of diabetic retinopathy
Source: Sci Rep. 2016 Dec 5;6:38445. doi: 10.1038/srep38445 (PMC5137162; doi:10.1038/srep38445)
Supplement: Supplementary Information [file srep38445-s1.pdf]

Supplementary information for

Basigin can be a therapeutic target to restore the retinal vascular barrier function in the mouse model of diabetic retinopathy

Mitsuru Arima, Dan Cui, Tokuhiro Kimura, Koh-Hei Sonoda, Tatsuro Ishibashi, Satoshi Matsuda, Eiji Ikeda

Supplementary Figure S1

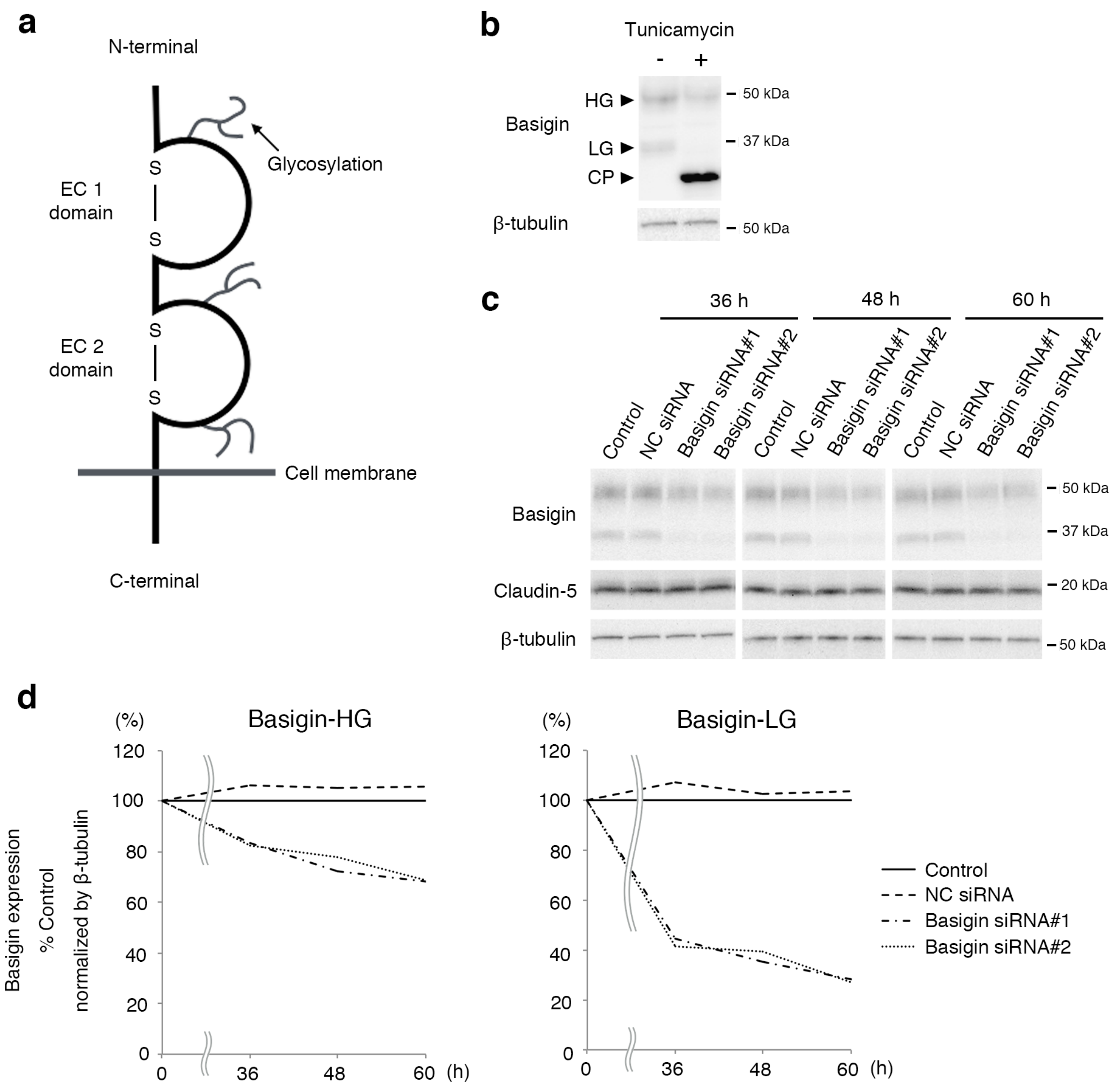

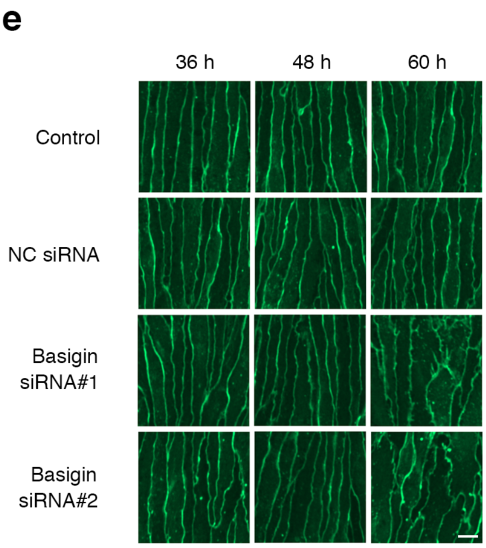

Supplementary Figure S1: Expression of basigin and claudin-5 in bEND.3 cells without and with the introduction of basigin siRNAs.

(**a**) Basigin structure. Basigin is transmembrane protein and has 3 glycosylated sites. Extracellular region is composed of 2 immunoglobulin-like domains (EC 1 and 2). (**b**) Western blot analyses for basigin and β-tubulin in bEND.3 cells. Two different sizes of basigin molecules are detected. They were confirmed, by addition of tunicamycin, to be a high glycosylation form (HG) and a low glycosylation form (LG), respectively. In addition to core protein (CP), basigin-HG remains even after treatment of tunicamycin probably due to its relatively long half-life. (**c**, **d**, and **e**) Western blot analyses for basigin, claudin-5 and β-tubulin (**c**), quantitative analyses of levels of basigin (**d**) and immunofluorescent images for claudin-5 (**e**) in cells at 36, 48, and 60 hours after transfection of basigin siRNAs. Suppression of basigin expression has no influence on claudin-5 expression. Pathological changes in cell morphology become detectable at 60 hours after induction of basigin siRNAs. S-S, disulphide bond; NC siRNA, non-silencing siRNA for negative control; Scar bar in (**d**), 10 µm.

Supplementary Figure S2

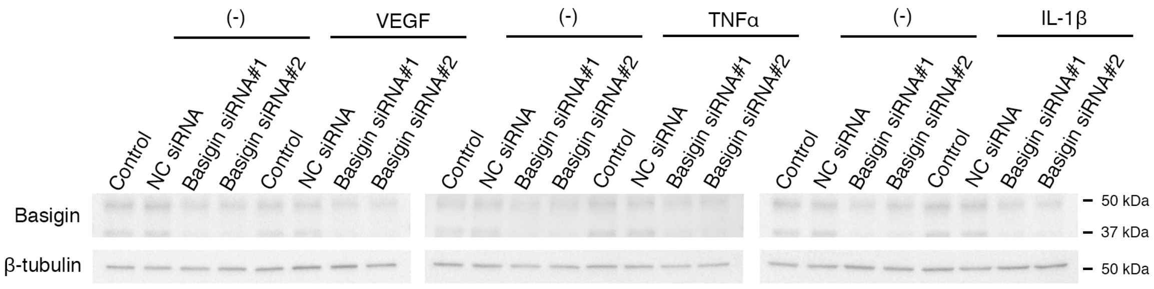

Supplementary Figure S2: Changes in the levels of basigin under inflammatory conditions.

Basigin expression is unchanged under stimulation with cytokines such as VEGF, TNFα and IL-1β. NC siRNA, non-silencing siRNA for negative control.

Supplementary Figure S3

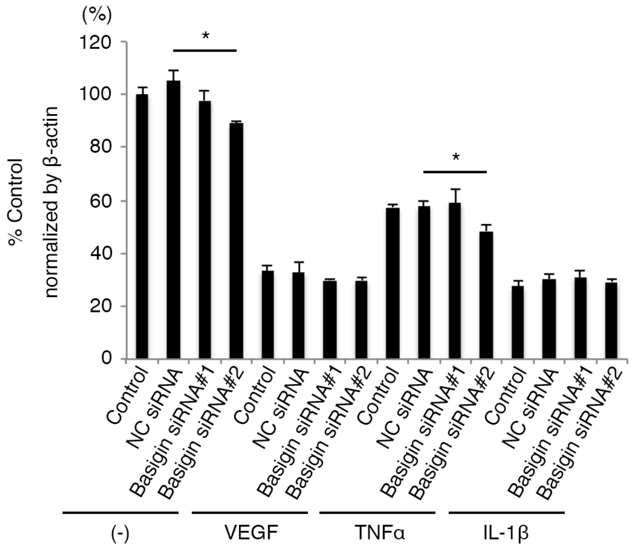

Supplementary Figure S3: Changes in mRNA levels of claudin-5 under inflammatory stimulation.

Suppression of basigin expression does not increase the claudin-5 mRNA levels in cells either without or with stimuli such as VEGF, TNFα and IL-1β, but rather decreases the levels of claudin-5 mRNA in cells without inflammatory stimuli as well as under the stimulus with TNFα. Error bars indicate s.d.. *$P < 0.01$; NC siRNA, non-silencing siRNA for negative control.

Supplementary Figure S4

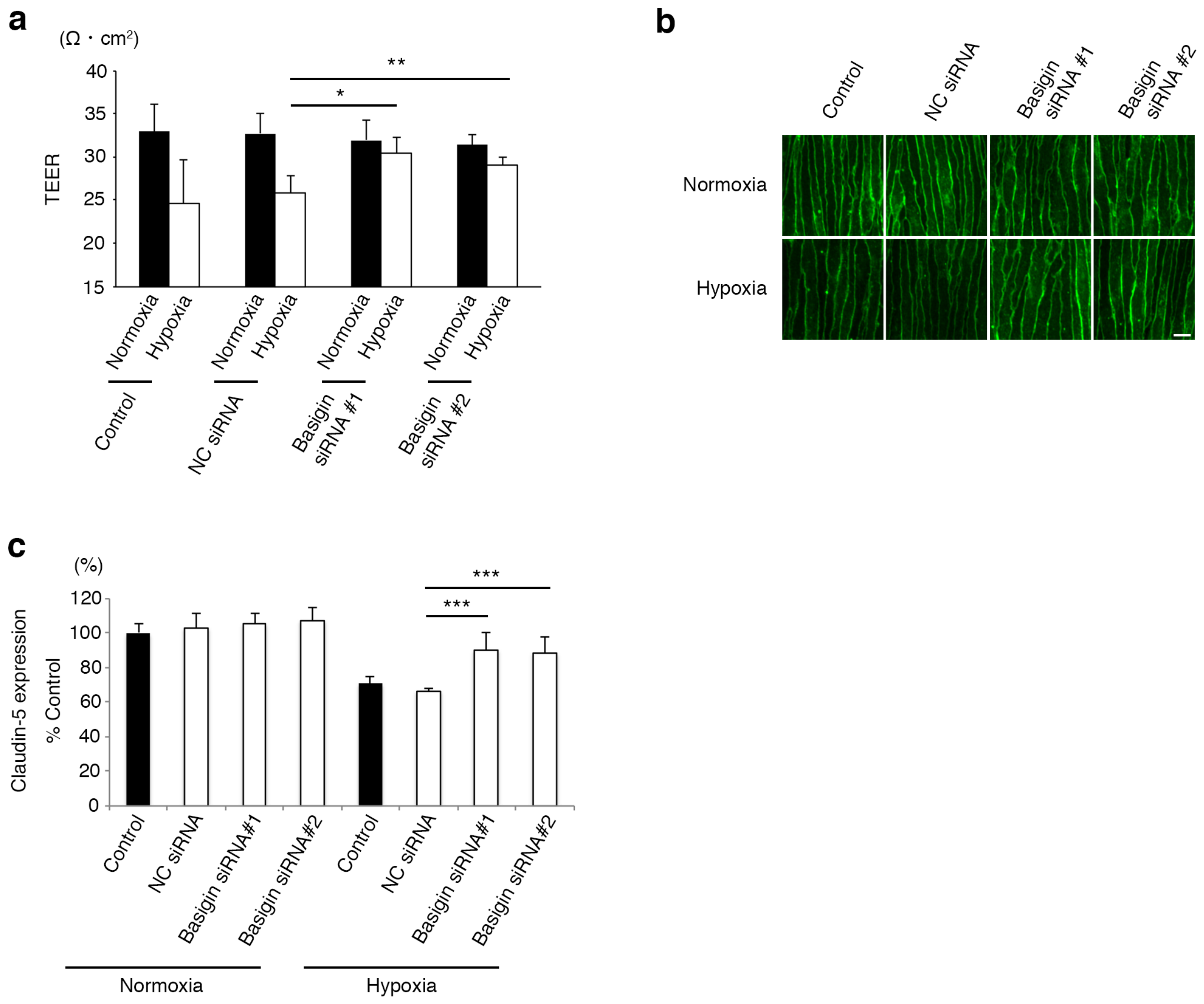

Supplementary Figure S4: Involvement of basigin in the impairment of neural vascular barrier under hypoxia.

(**a**) TEERs of bEND.3 monolayers under normoxia or hypoxia. bEND.3 monolayers under hypoxia are rescued from the decrease in TEERs by basigin knockdown. (**b**) Immunofluorescent images for claudin-5 under normoxia or hypoxia. Disappearance of claudin-5 from cell membranes in cells under hypoxia is suppressed by basigin knockdown. (**c**) Quantitative analyses of claudin-5 levels on cell membranes under normoxia or hypoxia. Claudin-5 levels in cells under hypoxia are significantly retained by basigin knockdown. Error bars indicate s.d.. *$P < 0.001$; **$P < 0.01$; ***$P < 0.05$; NC siRNA, non-silencing siRNA for negative control; Scar bar in (**b**), 10 μm.

Supplementary Figure S5

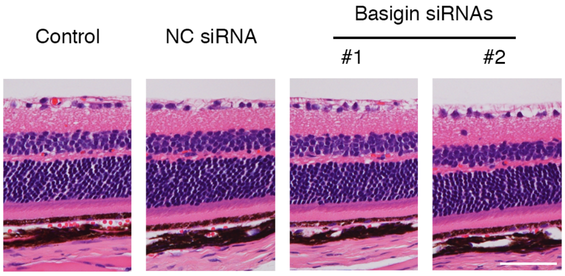

Supplementary Figure S5: Histology of retinal tissues without and with suppression of basigin expression for 3 days. No significant morphological changes are detectable. Experiments were performed 4 times. NC siRNA, non-silencing siRNA for negative control; Scar bar, 50 μm.

Supplementary Methods

Real-time quantitative polymerase chain reaction

Total RNA was extracted using RNeasy Mini Kit (Qiagen, Valencia, CA) and 1 μg of total RNA was reverse-transcribed using First Strand cDNA Synthesis Kit for RT-PCR (Roche) according to the manufacturer's instructions. Real-time quantitative polymerase chain reaction was performed with FastStart Essential DNA Green Master kit (Roche) on Light Cycler Nano (Roche). The thermal cycle conditions were set at 95°C for 10 minutes followed by 35 cycles at 95°C for 10 seconds, at 60°C for 10 seconds and at 72°C for 15 seconds. All values of mRNA were normalized to β-actin. The primers used were as follows: 5'- GGCACTCTTTGTTACCTTGACC-3' and 5'-CAGCTCGTACTTCTGTGACACC-3' for claudin-5; and 5'- GATGACCCAGATCATGTTTGA-3' and 5'-GGAGAGCATAGCCCTCGTAG-3' for β-actin. Experiments were performed independently in triplicate.

Hypoxic culture condition

Basigin siRNAs were transfected into bEND.3 cells at confluent state as described in Methods. After pre-incubation with basigin siRNAs for 36 hours, cells were cultured for 30 minutes in humidified incubators either with 5% CO_2 and 95% atmospheric air for normoxia and with 5% CO_2 and 1% O_2 balanced with N_2 for hypoxia. Oxygen-regulated Personal Multi Gas incubator (Astec Co., Ltd., Tokyo, Japan) was used to generate the hypoxic culture condition. TEER values were measured and immunocytochemical analyses were performed as described in Methods.
